# LncRNA HOTAIR promotes human liver cancer stem cell malignant growth through downregulation of SETD2

**DOI:** 10.18632/oncotarget.4443

**Published:** 2015-06-30

**Authors:** Haiyan Li, Jiahui An, Mengying Wu, Qidi Zheng, Xin Gui, Tianming Li, Hu Pu, Dongdong Lu

**Affiliations:** ^1^ School of Life Science and Technology, Tongji University, Shanghai 200092, China

**Keywords:** lncRNA HOTAIR, liver cancer stem cell, SETD2, DNA mismatch repair, microsatellite instability

## Abstract

Long non-coding RNA HOTAIR predicts negative tumor prognosis and exhibits oncogenic activity. Herein, we demonstrate HOTAIR promotes human liver cancer stem cell malignant growth through downregulation of SETD2. Mechanistically, HOTAIR reduces the recuritment of the CREB, P300, RNA polII onto the SETD2 promoter region that inhibits SETD2 expression and its phosphorylation. Thereby, the SETD2 binding capacity to substrate histone H3 is weakened, triggering a reduction of trimethylation on histone H3 thirty-sixth lysine, and thereby the H3K36me3–hMSH2-hMSH6-SKP2 complex is also decreased. Strikingly, the complex occupancy on chromosome is depressed, preventing from mismatch DNA repair. While reducing the degradation capacity of Skp_2_ for aging histone H3 bound to damaged DNA, the aging histone repair is impaired. Furthermore, that the damaged DNA escaped to repair can causes microsatellite instability(MSI) and abnormal expression of cell cycle related genes that may trigger the hepatocarcinogenesis. This study provides evidence for HOTAIR to promote tumorigenesis via downregulating SETD2 in liver cancer stem cells.

## INTRODUCTION

Long non-coding RNAs (lncRNAs) are in general considered as non-protein coding transcripts longer than 200 nucleotides which can target transcription factors, transcriptional activators or repressors, different components of the transcription reaction including RNA polymerase (RNAP) II and even the DNA duplex to regulate gene transcription and expression at the epigenetic level [1]. Moreover, lncRNAs played important roles in proliferation, apoptosis, tumorigenesis and its progression, invasiveness [2]. For example, metastasis-associated lung adenocarcinoma transcript 1 (MALAT1), prostate cancer associated non-coding RNA 1 (PRNCR1), prostate cancer gene expression marker 1 (PCGEM1), H19, and several new lncRNAs involve in glioma development [3]. Notably, the lncRNAs HOTTIP, H19, HOTAIR, MALAT1, antisense Igf2r (AIR), HOXA13, GTL2 (also called MEG3) and uc002mb have been reported in association with hepatocellular carcinoma (HCC) [4]. However, we are only beginning to understand the nature and extent of the involvement of lncRNAs on tumorigeneis.

It is worth noting that HOTAIR is associated with motility, invasion, and metastatic potential of cancer. HOTAIR is located within the Homeobox C (HOXC) gene cluster on chromosome 12 and is co-expressed with HOXC genes. HOTAIR is shuttled from chromosome 12 to chromosome 2 by a component of Polycomb Repressive Complex 2 (PRC2) which represses transcription of HOXD genes on chromosome 2 in trans. The Polycomb group (PcG) protein heterodimer EZH2-EED is necessary and sufficient for binding to the lncRNA HOTAIR [5]. HOTAIR recruits PRC2 to regulate chromosome occupancy by EZH2 (a subunit of PRC2), which leads to histone H3 lysine 27 trimethylation of the HOXD locus. It has been comfirmed that HOTAIR interacts with both PRC2 and lysine specific demethylase 1 (LSD1) complexes through its 5′ and 3′ domains, respectively, and serves as a scaffold to assemble PRC2 and LSD1 complexes to the HOXD gene cluster. It couples H3K27 methylation and H3K4 demethylation for epigenetic silencing of HOXD genes in multiple tissues. HOTAIR directly decreased WIF-1 expression by promoting its histone H3K27 methylation in the promoter region and then activated the Wnt/beta-catenin signaling pathway [6]. It has been demonstrated HOTAIR is pervasively overexpressed in most human cancers compared with noncancerous adjacent tissues [7]. HOTAIR expression is increased in pancreatic tumors compared with non-tumor tissue and is associated with more aggressive tumors [8]. Recent some studies revealed aberrant HOTAIR expression was associated with various sites of cancers such as breast, gastric, liver, lung, colorectal, pancreatic et al. and affected survival and prognosis of cancer patients [9]. HOTAIR plays a pivotal role in the development of gastric cancer [10]. HOTAIR contributes to the cisplatin resistance of lung adenocarcinoma (LAD) cells, at least in part, through the regulation of p21 expression [11]. Of interest, HOTAIR is capable of reprogramming chromatin organization and promoting cancer cell metastasis which involved in both esophageal squamous cell carcinoma (ESCC) progression and prognosis [12]. Notably, HOTAIR and XIST are targets of site-specific cytosine methylation. HOTAIR is a potential biomarker for ESCC prognosis, and the dysregulation of HOTAIR may play an important role in ESCC progression and survival [13, 14]. However, we are unclear for the effect mechanism of HOTAIR on tumorigeneis.

In this study, we identify that HOTAIR promotes human liver cancer stem cell malignant growth through downregulation of SETD2, the enzyme that trimethylates histone H3 lysine 36 (H3K36me3), is required for ATM activation upon DNA double-strand breaks (DSBs). It provides evidence for HOTAIR to play tumorigenesis roles via downregulating SETD2 in liver cancer stem cell, which may have potential therapeutic significance. HOTAIR may be a valuable predictor and a potential target for liver cancer prevention and treatment.

## RESULTS

### HOTAIR expression was negatively associated with the SETD2 expression in human liver cancer tissues

**Figure 1:**
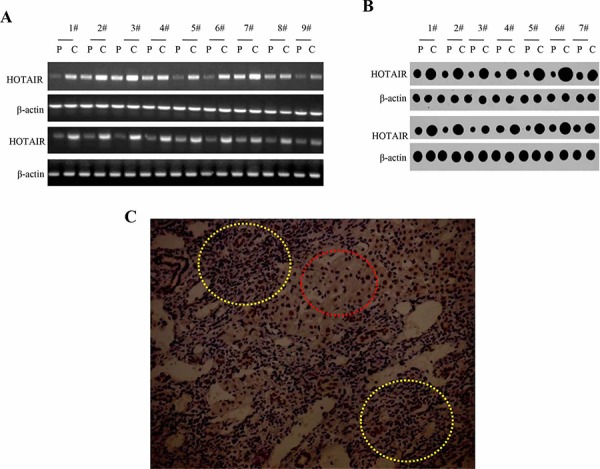
HOTAIR and SETD2 expression in human liver cancer tissue **A.** RT-PCR analysis of HOTAIR in liver cancer tissue (*C*) and its paracancerous liver tissues (*P*) respectively (indicated in upper). β-actin as internal control. **B.** Nuclear Run on analysis of HOTAIR in liver cancer tissue (*C*) and its paracancerous liver tissues (*P*) respectively (indicated in upper). β-actin as internal control. **C.** The representative analytic results of SETD2 immunohistochemistry staining of formalin-fixed, paraffin-embedded human liver cancer tissue (*indicated with yellow Dotted circles*) and their paired adjacent noncancerous tissues (*indicated with Dotted red circles*) from the same patient. (DAB stainning, original magnification × 100).

Given that cells lacking the SET Domain-Containing Protein 2 (a histone methyltransferase that is specific for lysine-36 of histone H3, SETD2) display microsatellite instability (MSI) and an elevated spontaneous mutation frequency, we consider whether the negative synergy between HOTAIR and SETD2 exists in hepatocarcinogenesis. To examine the relationship between long noncoding RNA HOTAIR and SETD2 in human primary liver cancer, we first detected the HOTAIR in 18 cases of human hepatocarocinoma tissues and their paired adjacent noncancerous tissues from the same patient by RT-PCR. The results showed that the HOTAIR level was significantly higher in human hepatocarocinoma tissues than in their paired adjacent noncancerous tissues (the upregulation expression rate 100%, *n* = 18, *P* < 0.01)(Figure 1A). Further, we preformed nuclear run on assay to detect the HOTAIR in 14 cases of human hepatocarocinoma tissues. The findings also showed that the HOTAIR was significantly higher in human hepatocarocinoma tissues than in their paired adjacent noncancerous tissues (the upregulation expression rate 100%, *n* = 14, *P* < 0.01)(Figure 1B). Then, we performed immunohistochemistry staining for SETD2 in formalin-fixed, paraffin-embedded 65 case of human hepatocarocinoma tissues and their paired adjacent noncancerous tissues(including aforementioned 18 cases human hepatocarocinoma tissues). The immunohistochemical detection showed reduced expression of SETD2 in hepatocarocinoma tissues compared with their paired adjacent noncancerous tissues(the downregulation expression rate 94.31%, *n* = 65, *P* < 0.01) (Figure 1C). In the 18 cases of human primary liver cancer, HOTAIR upexpression(100%) was negatively associated with the SETD2 down expression(100%) (Correlation coefficient, R = −1). Taken together, these results suggest there is negatively correlation between the HOTAIR upregulated expression and STED2 downregulated expression in human primary liver cancer.

### HOTAIR accelerates Human liver cancer stem cell (hLCSC) malignant proliferation

**Figure 2:**
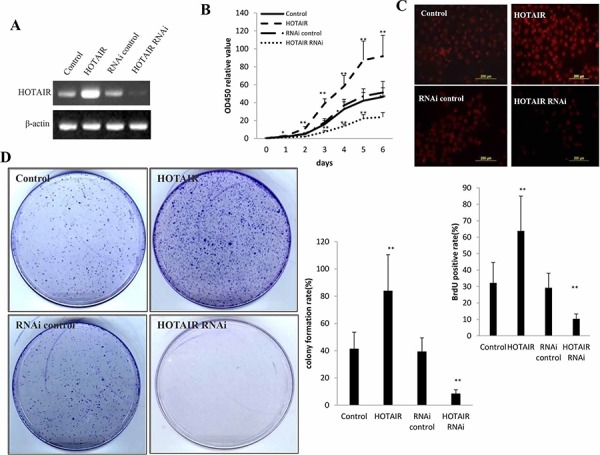
HOTAIR accelerlates human liver cancer stem cells (hLCSC) growth *in vitro* **A.** The RT-PCR analysis of HOTAIR in stable hLCSC cell lines transfected pCMV6-A-GFP, pCMV6-A-GFP-HOTAIR, pGFP-V-RS, pGFP-V-RS-HOTAIR.β-actin as internal control. **B.** Cells growth assay using CCK8. Each value was presented as mean ± standard error of the mean (SEM). **C.** S phase cells assay using BrdU. Each value was presented as mean ± standard error of the mean (SEM). **D.** Cells colony formation assay. Each value was presented as mean ± standard error of the mean (SEM).

To address whether the HOTAIR influences on primary liver cancer cells malignant proliferation, we established the stable human liver cancer stem cell (hLCSC) cell lines transfected with pCMV6-A-GFP, pCMV6-A-GFP-HOTAIR, pGFP-V-RS, pGFP-V-RS-HOTAIR respectively. We confirmed HOTAIR was significantly overexpressed in pCMV6-A-GFP-HOTAIR transfected hLCSC compared with control, while HOTAIR was significantly knocked down in pGFP-V-RS-HOTAIR transfected hLCSCs compared the control (Figure 2A). At the First time, we detected these cells proliferation *in vitro*. As shown in Figure 2B, HOTAIR overexpression enhanced the and HOTAIR knockdown inhibited hLCSC proliferation ability (*P* < 0.01). Next, we detected the S phase cells by BrdU staining in HOTAIR overexpression or knockdown hLCSCs. The BrdU staining findings showed that the BrdU positive rate added up to 63.8% in HOTAIR overexpressed hLCSCs, while the BrdU positive rate added up to 32.2% in control (*p* < 0.01). On the other hand, the BrdU positive rate added up to 10.3% in HOTAIR knocked-down hLCSCs, while The BrdU positive rate added up to 29.2% in RNAi control hLCSCs (*P* < 0.01) (Figure 2C). Then we conducted cell colony-formation efficiency assay in these hLCSCs. The colony-formation rate added up to 83.8% in HOTAIR overexpressed hLCSCs, while the colony-formation rate added up to 41.2% in control (*p* < 0.01). Moreover, the colony-formation rate added up to 8.3% in HOTAIR knocked-down hLCSC, while the colony-formation rate added up to 39.2% in RNAi control hLCSCs. (*P* < 0.01) (Figure 2D). Taken together, these results suggest that long noncoding RNA HOTAIR accelerates the liver cancer stem cells proliferation *in vitro*.

### HOTAIR promotes liver cancer stem cells growth *in vivo*

**Figure 3:**
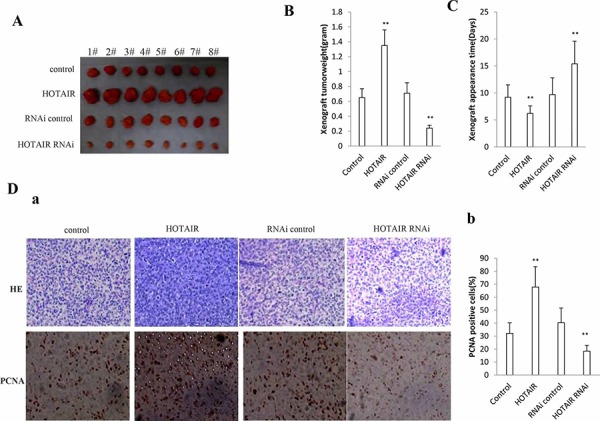
HOTAIR promotes human liver cancer stem cells (hLCSCs) growth *in vivo* **A.** The mice were stratified and the tumors were recovered. The photography of xenograft tumor in the four groups (indicated in left). **B.** The wet weight of each tumor was determined for each mouse. Each value was presented as mean ± standard error of the mean (SEM). **C.** The Xenograft appearance time (days). Each value was presented as mean ± standard error of the mean (SEM). **D.** a. A portion of each tumor was fixed in 4% paraformaldehyde and embedded in paraffin for histological hematoxylin-eosin (HE) staining (*upper*) and anti-PCNA immunostainning (*lower*). (original magnification × 100). b. PCNA positive cells analysis. Each value was presented as mean ± standard error of the mean (SEM).

To test the effect of HOTAIR on tumorigenesis *in vivo*, the hLCSCs stable cell lines with altered expression of HOTAIR were injected subcutaneously into Balb/C (severe combined immunodeficiency) mice. As shown in Figure 3A, when HOTAIR was overexpressed, the xenograft tumor weight increased approximately two folds when compared to the corresponding control group (1.35 ± 0.39 grams versus 0.65 ± 0.12 grams, *P* < 0.01). On the other hand, when HOTAIR was knocked down, the average xenograft tumor weight decreased to approximately one third of the control weight (0.71 ± 0.13 grams versus 0.24 ± 0.06 grams, *P* < 0.01) (Figure 3B). HOTAIR overexpression resulted in early xenograft tumor formation compared to the control group (6.21 ± 1.61 days versus 9.23 ± 2.01 days, *P* < 0.05). In contrast, the time of xenograft tumor appearance was prolonged in the HOTAIR knockdown group compared to the control group (15.41 ± 4.12 days versus 9.74 ± 3.14 days, *p* < 0.01) (Figure 3C). Pathological picture (HE stain) of xenograft tumor showed that tumor tissue possessed more poor-differentiation cells and less moderately or well-differentiation cells in HOTAIR overexpression group than that of control group, and less poor-differentiation cells and more moderately or well-differentiation cells in HOTAIR knockdown group than that of control group (Figure 3Da). The proliferation index (calculated as percentage of PCNA-positive cells)was significantly higher in HOTAIR overexpressed tumors compared to the vector control (67.82 ± 13.97% versus 32.14 ± 7.82, *p* < 0.01). Conversely, the percentage of PCNA positive cells was significantly lower in HOTAIR knockdown tumors (18.41 ± 4.23% versus 40.65 ± 9.86%, *p* < 0.01) (Figure 3Db). Taken together, these findings demonstrate that HOTAOIR enhances HCC malignant progression *in vivo*.

### HOTAIR inhibits SETD2 expression and its phosphorylation

**Figure 4:**
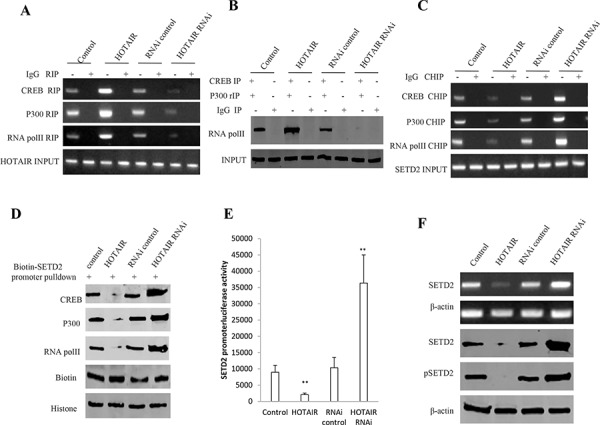
HOTAIR inhibits SETD2 expression in human liver cancer stem cell (hLCSC) **A.** RNA Immunoprecipitation (RIP) with anti-CREB, anti-P300 and anti-RNApolII followed by RT-PCR with HOTAIR primers in HOTAIR overexpression or knockdown human liver cancer stem cells (hLCSC). IgG RIP as negative control. HOTAIR as INPUT. B. anti-CREB Co-Immunoprecipitation (IP) and anti-P300 repeated Co-Immunoprecipitation (rIP) followed by Western blotting with anti-RNA polII. IgG IP as negative control. INPUT refers to Western blotting with anti-RNA polII. **B.** HOTAIR inhibits SETD2 expression in human liver cancer stem cell (hLCSC). **C.** Western blotting with anti-H3K36me1, anti-H3K36me2, anti-H3K36me1. β-actin as internal control. **C.** Chromatin Immunoprecipitation (CHIP) with anti-CREB, anti-P300 and anti-RNApolII followed by PCR with SETD2 promoter primers in HOTAIR overexpression or knockdown hLCSC cell line. IgG CHIP as negative control. SETD2 promoter DNA as INPUT. **D.** Biotin-SETD2 promoter probe pulldown followed by Western blotting anti-CREB, anti-P300 and anti-RNApolII primers in HOTAIR overexpression or knockdown human liver cancer stem cell (hLCSC). Biotin as INPUT and Histone as internal control. **E.** SETD2 promoter luciferase activity assay in HOTAIR overexpression or knockdown human liver cancer stem cell (hLCSC). Each value was presented as mean ± standard error of the mean (SEM). **F.** SETD2 expression analysis by RT-PCR with SETD2 cDNA primers and Western blotting with anti-SETD2 and anti-pSETD2. β-actin as internal control.

SETD2 protein is a histone methyltransferase that is specific for lysine-36 of histone H3, and methylation of this residue is associated with active chromatin. SETD2 protein also contains a novel transcriptional activation domain and has been found associated with hyperphosphorylated RNA polymerase II [15]. Given that there was negatively correlation between the expression of HOTAIR and STED2 in human primary liver cancer, we had reasons to consider whether HOTAIR influence on the SETD2 expression and its modification. SETD2 promoer region contains a CREB bound element that is in favor of RNA polII activity. The recruitment of CREB-P300-RNA polII complex to the SETD2 promoter may be blocked by HOTAIR. At the first time, we performed the RNA Immunoprecipitation (RIP) with anti-CREB, anti-P300 and anti-RNApolII. Our results showed that HOTAIR overexpression increased and HOTAIR knockdown decreased the interplay between HOTAIR and CREB, P300, RNApolII in hLCSCs cell lines respectively (Figure 4A). Then, Repeat-Immunoprecipitation results showed that HOTAIR overexpression increased and HOTAIR knockdown decreased the interplay among CREB, P300, RNA polII in hLCSCs respectively. Next, we adopted chromatin Immunoprecipitation (CHIP) with anti-CREB, anti-P300 and anti-RNApolII. We clearly found that HOTAIR overexpression decreased and HOTAIR knockdown increased the occupancy of CREB, P300, RNA polII on STED2 promoter region in hLCSCs respectively (Figure 4B). Further on, we completed the biotin-SETD2 promoter pulldown in these hLCSCs. As shown in Figure 4C, HOTAIR overexpression decreased and HOTAIR knockdown increased the binding of CREB, P300, RNApolII to SETD2 promoter probe in hLCSCs respectively (Figure 4D). Therefore, we speculated HOTAIR competitively blocked the loading of CREB, P300, RNApolII on SETD2 promoter region. Moreover, HOTAIR overexpression also decreased and HOTAIR knockdown also increased SETD2 promoter luciferase activity in hLCSCs (Figure 4E). Of significance, the results showed that HOTAIR overexpression reduced and HOTAIR knockdown enhanced the SETD2 expression and its phosphorylation modification (Figure 4F). Taken together, these findings suggest HOTAIR inhibited the SETD2 expression through blocking RNApolII catalytic function by dissociating the CREB, P300, RNApolII complex for SETD2 transcription.

### HOTAIR reduces the hMSH2/6 binding to H3k36me3 and Skp2

**Figure 5:**
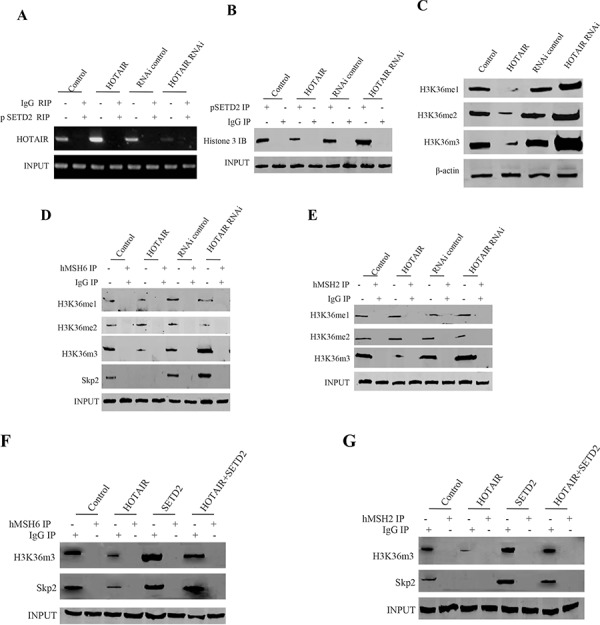
The HOTAIR inhibits the interaction among hMSH6, H3k36me3 and Skp2 in stable human liver cancer stem cell (hLCSC) transfected with pCMV6-A-GFP, pCMV6-A-GFP-HOTAIR, pGFP-V-RS, pGFP-V-RS-HOTAIR **A.** RNA Immunoprecipitation (RIP) with anti-pSETD2 followed by RT-PCR with HOTAIR primers in HOTAIR overexpression or knockdown human liver cancer stem cells (hLCSC). IgG RIP as negative control. HOTAIR as INPUT. **B.** anti-SETD2 Co-Immunoprecipitation (IP) followed by Western blotting with anti-Histone3. IgG IP as negative control. INPUT refers to Western blotting with anti-SETD2. **C.** Western blotting with anti-H3K36me1, anti-H3K36me2, anti-H3K36me1. β-actin as internalcontrol. **D.** Anti-hMSH6 Co-Immunoprecipitation (IP) followed by Western blotting with anti-H3K36me3, anti-H3K36me2, anti-H3K36me1 anti-SKP2. IgG IP as negative control. INPUT refers to Western blotting with anti-hMSH6. **E.** Anti-hMSH2 Co-Immunoprecipitation (IP) followed by Western blotting with anti-H3K36me3, anti-H3K36me2, anti-H3K36me1. IgG IP as negative control. INPUT refers to Western blotting with anti-hMSH2. **F.** Anti-hMSH6 Co-Immunoprecipitation (IP) followed by Western blotting with anti-H3K36me3, anti-SKP2. IgG IP as negative control. INPUT refers to Western blotting with anti-hMSH6. **G.** Anti-hMSH2 Co-Immunoprecipitation (IP) followed by Western blotting with anti-H3K36me3, anti-SKP2. IgG IP as negative control. INPUT refers to Western blotting with anti-hMSH2.

It has been demonstrated that the ternary complexes hMSH6-H3k36me3-Skp2 plays an important role in the injury DNA and old protein repair [16]. In order to address whether the HOTAIR influences on the formation of the complexes, we first have reasonable to consider to identify HOTAIR influence on the modification of three methylation on histone 3 thirty-sixth lysine. At the first time, we performed the RNA Immunoprecipitation(RIP) with anti-pSTED2 and the findings showed that the binding capacity of pSTED2 to HOTAIR increased in HOTAIR overexpressed hLCSCs and reduced in HOTAIR knocked down hLCSCs (Figure 5A). Next, we performed CO-IP experiment in hLCSCs. As shown in Figure 5B, the overexpression of HOTAIR inhibited the interpay between pSETD2 and Histone H3, while the knockdown of HOTAIR enhanced the interplay between SETD2 and Histone H3. Importantly, the findings identified that the overexpression of HOTAIR decreased the level of H3K36me1, H3K36me2, H3K36me3, while the knockdown of HOTAIR increased the level of H3K36me1, H3K36me2, H3K36me3 in hLCSCs (Figure 5C). Further analysis indicated that the overexpression of HOTAIR inhibited the interplay among hMSH6, H3Kme3 and Skp2, while the knockdown of HOTAIR promoted the interplay among hMSH6, H3K36me3, Skp2 in hLCSCs (Figure 5D). Similary, our results showed that the overexpression of HOTAIR inhibited the interplay between hMSH2, H3K36me3, while the knockdown of HOTAIR promoted the interplay between hMSH2 and H3K36me3 in hLCSCs (Figure 5E). However, as shown in Figure 5F, the overexpression of HOTAIR inhibited the interplay among hMSH6, H3Kme3 and Skp2, while the HOTAIR overexpression plus STED2 overexpression did not alter the interplay among hMSH6, H3K36me3, Skp2 in hLCSCs. As shown in Figure 5G, the overexpression of HOTAIR inhibited the interpay among hMSH2, H3K36me3 and Skp2, while the HOTAIR onerexpression plus STED2 overexpression did not alter the interplay among hMSH2, H3K36me3 and Skp2 in hLCSCs. Taken together, our results underline that HOTAIR inhibited the level of H3K36me3, and therefore reduced the formation of hMSH6-hMSH2-H3k36me3-Skp2 complex.

### HOTAIR inhibits the DNA damage repair

**Figure 6:**
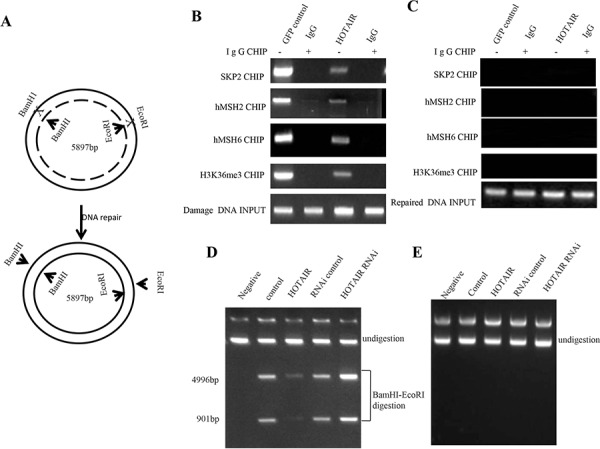
The HOTAIR inhibits the hMSH2/6-H3k36me3-Skp2 ternary complex loading onto the injury DNA in human liver cancer stem cell (hLCSC) **A.** The schematic illustrates a model that injury plasmids (EcoRI mismatch) was repaired after the plasmid was transfected and intergrated into chromosome. **B.** Chromatin Immunoprecipitation (CHIP) with anti-.SKP2, anti-hMSH2, anti-hMSH6, anti-H3K36me3 followed by PCR with damaged DNA primers. IgG CHIP as negative control. damaged DNA as INPUT. **C.** Chromatin Immunoprecipitation (CHIP) with anti-SKP2, anti-hMSH6, anti-hMSH6, anti-H3K36me3 followed by PCR with match double DNA primers. IgG CHIP as negative control. match double DNA DNA as INPUT. **D.** Restriction endonuclease analysis with BamHI and EcoRI for DNA injury repair. **E.** Restriction endonuclease analysis with BamHI and EcoRI for DNA injury imrepair.

DNA mismatch repair (MMR) ensures replication fidelity by correcting mismatches generated during DNA replication. An epigenetic histone mark, H3K36me3, is required *in vivo* to recruit the mismatch recognition protein hMutSα (hMSH2-hMSH6) onto chromatin [16]. To address whether HOTAIR influenced on DNA damage repair by downregulated H3K36me3, we constructed a mismatch plasmids (EcoRI mismatch) which could be repaired after the plasmid was transfected and intergrated into chromosome (Figure 6A). After transfected with the plasmid, we cperformed the CHIP assay in HOTAIR overexpressed hLCSCs. The results revealed that HOTAIR overexpression inhibited the loading of Spk2, H3k36me3, hMSH2, hMSH6 onto the mismatch DNA (Figure 6B). Strikingly, the occupancy of Spk2, H3k36me3, hMSH2, hMSH6 on the match DNA was not been displayed in the HOTAIR overexpressed hLCSCs (Figure 6C). Next, we performed restriction endonuclease analysis for DNA injury repair with BamHI and EcoRI. As shown in Figure 6D, HOTAIR overexpression significantly reduced the BamHI and EcoRI restriction product, while HOTAIR knockdown significantly increased the BamHI and EcoRI restriction product. However, the negative control did not show the similar effect (Figure 6E). Obviously, these observations, taken together, suggest that the HOTAIR make concerted efforts to prevent the hMSH2/6-H3k36me3-Skp2 complex from occupancy on the site of DNA damage which impedes DNA damage repair.

### HOTAIR enhances microsatellite instability (MSI) and triggers gene abnormral expression

**Figure 7:**
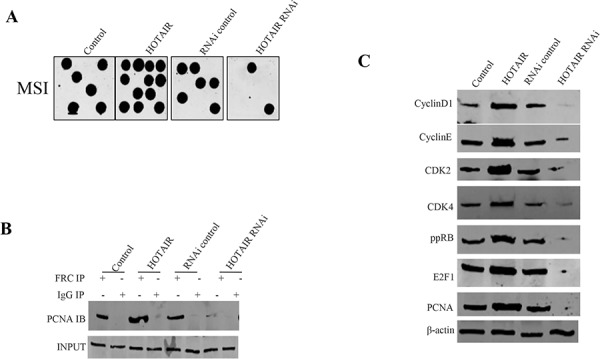
HOTAIR triggers MSI and abnormral gene expression in stable human liver cancer stem cell (hLCSC) transfected with pCMV6-A-GFP, pCMV6-A-GFP-HOTAIR, pGFP-V-RS, pGFP-V-RS-HOTAIR **A.** Microsatallite Instability (MSI) analysis through Dot blot (Slot blot) using various Biotin labling MSI probes (Biotin-MSIs). **B.** Co-immunoprecipitation (IP) with RFC followed by Western blotting with anti-PCNA. Anti-RFC western blotting as INPUT. **C.** Western blotting with anti-CyclinD1, anti-CyclinE, anti-CDK2, anti-CDK4, anti-ppRB, anti- E2F1, anti-PCNA.β-actin as internal control.

It is a fact that cells lacking the H3K36 trimethyltransferase SETD2 display microsatellite instability (MSI) which may alter cell cycle protein expression. We performed a microsatellite instability (MSI) assay through Dot blot (Slot blot) using various Biotin labling MSI probes (Biotin-MSIs) in hLCSCs cells transfected with pCMV6-A-GFP, pCMV6-A-GFP-HOTAIR, pGFP-V-RS, pGFP-V-RS-HOTAIR. As shown in Figure 7A, HOTAIR overexpression increased the MSI compared to control, while HOTAIR knockdown reduced the MSI compared to control. Co-IP results showed that HOTAIR overexpression increased the interaction between RFC and PCNA compared to control, while HOTAIR knockdown reduced interaction between RFC and PCNA compared to control (Figure 7B). Western blotting results showed that the overexpression of HOTAIR increased the expression of CyclinE, CyclinD1, CDK2, CDK4, ppRB, E2F1, PCNA, while the knockdown of HOTAIR reduced the expression of CyclinE, CyclinD1, CDK2, CDK4, ppRB, E2F1, PCNAs (Figure 7C). Taken together, these results suggest HOTAIR causes microsatellite instability (MSI) and abnormral expression of cell cycle related gene, e.g. CDK2, CyclinE, CDK4, CyclinD1, PCNA, ppRB, E2F1.

### SETD2 activity is crucial for oncogenic HOTAIR actions

**Figure 8:**
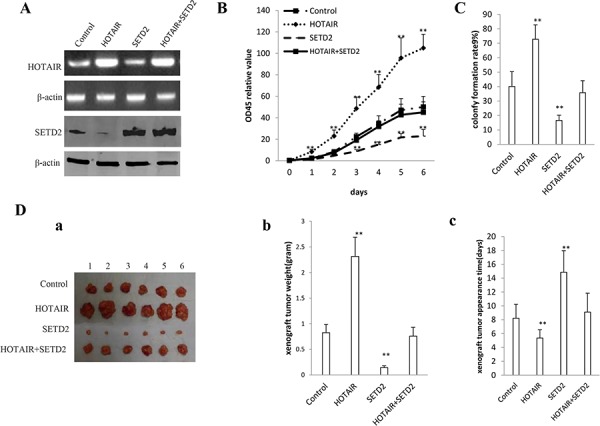
The rescued experiment of carcinogenesis effect of the HOTAIR SETD2 overexpression abrogated the HOTAIR oncogenic function in stable human liver cancer stem cell (hLCSC) transfected with pCMV6-A-GFP, pCMV6-A-GFP-HOTAIR, pcDNA3.1-SETD2, pCMV6-A-GFP-HOTAIR plus pcDNA3.1-SETD2. **A.** The RT-PCR analysis of HOTAIR (*upper*) and the western blotting analysis with anti-SETD2 (*lower*). β -actin as internal control. **B.** Cells growth assay using CCK8. Each value was presented as mean ± standard error of the mean (SEM). **C.** Cells colony-formation efficiency assay. Each value was presented as mean ± standard error of the mean (SEM). **D.** *In vivo* test in human liver cancer stem cells (hLCSC) transfected with pCMV6-A-GFP, pCMV6-A-GFP-HOTAIR, pcDNA3.1-SETD2, pCMV6-A-GFP-HOTAIR plus pcDNA3.1-SETD2. a. The mice were stratified and the tumors were recovered. The photography of xenograft tumor in the three groups (indicated in *left*). b. The wet weight of each tumor was determined for each mouse. Each value was presented as mean ± standard error of the mean (SEM). c. The xenograft appearance time (day). Each value was presented as mean ± standard error of the mean (SEM).

To explicit whether HOTAIR oncogenic function was controled by SETD2, we performed rescue test in the HOTAIR overexpressed hLCSCs cells, including control, HOTAIR overexpression, SETD2 overexpression, HOTAIR overexpression plus SETD2 overexpression. First, we performed RT-PCR analysis for HOTAIR and Western blotting analysis for SETD2 in hLCSCs cells. As shown in Figure 8A, HOTAIR was overexpressed in hLCSCs with HOTAIR overexpression, HOTAIR overexpression plus SETD2 overexpression. SETD2 was overexpressed in hLCSCs with SETD2 overexpression, HOTAIR overexpression plus SETD2 overexpression. On the contrary, SETD2 expression was reduced in HOTAIR overexpression hLCSCs. Next, we conducted cells proliferation CCK8 assay *in vitro* in hLCSCs with control, HOTAIR overexpression, SETD2 overexpression and HOTAIR overexpression plus SETD2 overexpression. HOTAIR overexpression promoted and SETD2 inhibited the hLCSCs proliferation, while HOTAIR overexpression plus SETD2 overexpression abrogated the HOTAIR action (Figure 8B). In cell colony-formation efficiency assay, we clearly found that cell colony-formation rate was significantly higher in HOTAIR overexpression group (72.77 ± 9.98%) and lower in SETD2 overexpresion group (16.58 ± 3.61%) than in control group (40.07 ± 10.31%) respectively (*P* < 0.01), however, the cell colony-formation rate was significantly no difference in HOTAIR overexpression plus SETD2 overexpression (35.77 ± 8.33%) compared to control group (40.07 ± 10.31%) (*P* > 0.05) (Figure 8C). Further, we performed *in vivo* test in these HOTAIR and/or SETD2 overexpressed hLCSCs. As shown in Figure 8Da, HOTAIR overexpression promoted and HOTAIR knockdown inhibited the xenograft tumor formation, while HOTAIR overexpression plus SETD2 overexpression did not altered the xenograft tumor growth respectively. The average wet weight of xenograft tumors were significantly increased in HOTAIR overexpression group (2.35 ± 0.37 gram) and decreased in SETD2 overexpression group (0.14 ± 0.037 gram) compared to control (0.825 ± 0.163 gram), while The average wet weight of xenograft tumors were significantly not altered in HOTAIR overexpression plus SETD2 overexpression group (0.76 ± 0.17 gram) compared to control (0.825 ± 0.163 gram) (*P* > 0.05) (Figure 8Db). The average tumor appearance time were significantly decreased in HOTAIR overexpression group (5.34 ± 1.19 days) and increased in STED2 overexpression group (14.85 ± 3.12 days) compared to control (8.22 ± 2.02 days), while the average tumor appearance time were significantly no difference in HOTAIR overexpression plus SETD2 overexpression group (9.1 ± 2.73 days) compared to control (8.22 ± 2.02 days) (*P* > 0.05) (Figure 8Dc). Taken together, these observations suggest that the overexpressed HOTAIR oncogenic action was abrogated by the overexpression of SETD2 in human liver cancer stem cells.

## DISCUSSION

**Figure 9:**
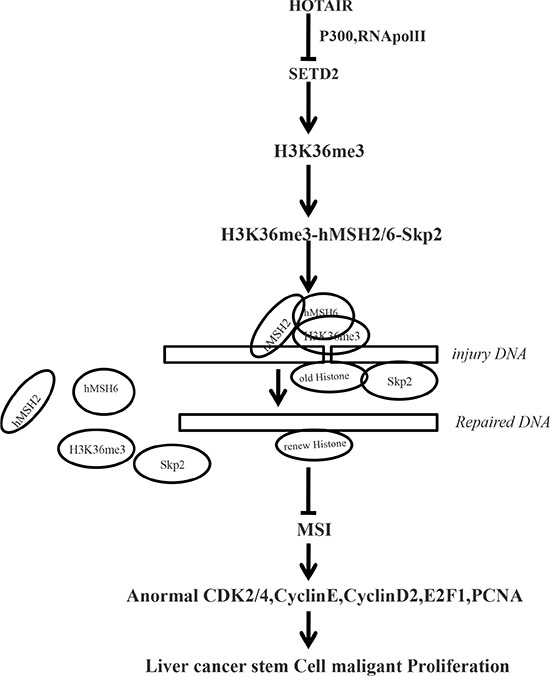
The schematic illustrates a model that HOTAIR promotes human liver cancer stem cell (hLCSC) malignant transformation through downregulation of SETD2 Oncogenic HOTAIR reduces the recuritment of the CREB, P300, RNApolII onto the SETD2 promoter region that inhibits SETD2 expression and its phosphorylation. The reduced SETD2 binding capacity of substrate histone, triggering a reduction of tri-methylation on histone 3 thirty-sixth lysine, and thereby the H3K36me3–hMSH2-hMSH6-SKP2 four complex is reduced. And the four complex occupancy on chromosome is reduced, preventing from damage DNA repair. While reducing the degradation ability, of SKP2 for old histone H3 bound to damaged DNA, the aging Histone repair is delayed. That the damaged DNA escaped to repair can lead to microsatellite instability (MSI) and abnormal expression of cell cycle related genes that promotes hepatocarcinogenesis.

It has been confirmed that a class of lncRNAs are dysregulated in hepatocellular carcinoma(HCC) and closely related with tumorigenesis, metastasis, prognosis or diagnosis [17]. Our studies are now indicated to evaluate the effects of HOTAIR in liver cancer stem cells (Figure 9). To our knowledge, this is the first report demonstrating HOTAIR plays a positive role in liver carcinogenesis through the cascade of HOTAIR-SETD2-H3K36me3-hMSH2/6-Skp2 signaling.

To date, accumulating evidence indicates that HOTAIR plays a critical role in cancer progression and metastasis. HOTAIR upregulation was associated with larger tumor size, advanced pathological stage and extensive metastasis, and also correlated with shorter overall survival of gastric cancer, endometrial cancer, lung cancer, renal cell carcinoma (RCC) patients [18, 19, 20, 21]. HOTAIR overexpression promoted the proliferation, migration and invasion of gastric carcinoma cells, endometrial cancer, lung cancer, renal cell carcinoma (RCC) [22, 23, 24, 25]. Enforced expression of HOTAIR in epithelial cancer cells induced genome-wide re-targeting of Polycomb repressive complex 2 (PRC2) to an occupancy pattern more resembling embryonic fibroblasts, leading to alter histone H3 lysine 27 methylation, gene expression, and increased cancer invasiveness and metastasis [26]. Intriguingly, HOTAIR could promote migration and invasion of hepatocellular carcinoma cells by inhibiting RBM38 [27] and activating the Wnt/β-catenin signaling pathway [28]. Herein, the involvement of HOTAIR promotion of liver cancer stem cell growth is supported by results from two parallel sets of experiments: (1) we clearly reveal that HOTAIR is overexpressed in human liver cancer tissue; (2) HOTAIR promotes human liver cancer stem cell malignant growth in *vitro* and in *vivo*. Evidently, our observations demonstrated that HOTAIR is crucial for cell growth and viability in liver cancer stem cells. According to the aforementioned findings and reorts, HOTAIR is a strong oncogenic long noncoding RNA.

Our data suggest that HOTAIR reduced the SETD2 expression on the transcriptional level through blocking RNApolII catalytic function by dissociating the CREB-P300- RNApolII complex. SETD2 protein is a histone methyltransferase that is specific for lysine-36 of histone H3, and methylation of this residue is associated with active chromatin. SETD2 protein also contains a novel transcriptional activation domain and has been found associated with hyperphosphorylated RNA polymerase II [29]. The SETD2 gene has been shown to play a tumour suppressor role in human cancer [30]. DNA mismatch repair (MMR) ensures replication fidelity by correcting mismatches generated during DNA replication. SETD2 is required for ATM activation upon DNA double-strand breaks (DSBs) and homologous recombination repair of DSBs by promoting the formation of RAD51 presynaptic filaments [31, 32, 33]. Depletion of SETD2 severely impedes homologous recombination (HR) at such DNA double-strand breaks (DSBs) [34]. DNA mismatch repair (MMR) maintains genomic integrity by correction of mispaired bases and insertion-deletion loops. The MMR pathway can also trigger a DNA damage response upon binding of MutSα to specific DNA lesions [35]. Elevated mutation rates (mutator phenotype), including simple repeat instability [microsatellite instability (MSI)] are a signature of MMR defects [36]. Especifically, Cells lacking the H3K36 trimethyltransferase SETD2 display microsatellite instability (MSI) and an elevated spontaneous mutation frequency [16]. Accordingly, SETD2 is a vital target of HOTAIR which involves in DNA damage repair epigenetically. Therefore, HOTAIR greatly impairs the DNA damage repair capacity and increases microsatellite instability (MSI) in liver stem cells.

Although reduction of SETD2 may partly contribute to HOTAIR-medicated promotion of liver cancer stem cell growth, our findings in this study provide novel evidence for an active role of SETD2 in HOTAIR-mediated promotion of liver cancer stem cell growth. This assertion is based on several observations: (1) There is negatively correlation between the HOTAIR upregulated expression and SETD2 downregulated expression in human primary liver cancer. (2) overexpression of SETD2 prevented HOTAIR-induced promotion of liver cancer stem cell proliferation, colony formation and growth *in vivo*; (3)SETD2 knockdown prevented HOTAIR depletion-mediated decrease of cell proliferation, colony formation and migration. (4) The overexpressed HOTAIR oncogenic action was abrogated by the overexpression of SETD2 in human liver cancer stem cells. These findings are noteworthy, given that SETD2 is a and functions as a key tumor suppressor mediate various biological processes including cell proliferation, differentiation.

Our findings clearly showed that HOTAIR reduced H3k36me3 by inhibiting SETD2 expression. Trimethylation of Lys36 in histone H3 (H3K36me3) is deposited onto the nucleosomes in the transcribed regions after RNA polymerase II elongation [37]. H3K36me3 coordinates events associated with the elongation phase of transcription and is also emerging as an important epigenetic regulator of cell growth and differentiation [38]. Disruption of the SETD2-H3K36me3 pathway is a distinct epigenetic mechanism for tumorigenesis development [39]. Strikingly, H3K36me3 is required *in vivo* to recruit the mismatch recognition protein hMutSα (hMSH2-hMSH6) onto chromatin through direct interactions with the hMSH6 PWWP domain [16]. The abundance of H3K36me3 in G1 and early S phases ensures that hMutSα is enriched on chromatin before mispairs are introduced during DNA replication. Reducing H3K36me3 levels by overexpressing KDM4A/JMJD2A also reduces HR repair events [40].

Strikingly, we also demonstrated that HOTAIR decreased the interaction among MSH2, MSH6, H3K36me, Skp2 which hinders the DNA injury repair. The human MSH2/6 complex is essential for mismatch recognition during the repair of replication errors. hMSH2/6 formed a complex with BLM-p53-RAD51 in response to the damaged DNA forks during double-stranded break repair [41]. MSH6 is a key protein in MMR-dependent DNA damage response and communication with other DNA repair pathways within the cell. MSH6 is unstable in the absence of MSH2, however it is the DNA lesion-binding partner of this heterodimer [42, 43]. hMSH2-hMSH6 sliding clamps trap localized fluctuations in nucleosome positioning and/or wrapping that ultimately leads to disassembly, and highlight unanticipated strengths of the Molecular Switch Model for mismatch repair (MMR) [44]. Our observations identify HOTAIR inhibits the hMSH2/6-H3k36me3-Skp2 complex occupancy on the site of DNA damage and impede DNA damage repair.

Of Significance, histone repair is absorbing and of great concern. We observed the histone repair phenomenons and reveal that Skp2 degrades the old histone replaced by novel histone which is very important for DNA repair course. *Skp2* gene encodes a member of the F-box protein family which is characterized by an approximately 40 amino acid motif, the F-box. The F-box proteins constitute one of the four subunits of ubiquitin protein ligase complex called SCFs (SKP1-cullin-F-box), which function in phosphorylation-dependent ubiquitination.

Further on, increased Microsatellite instability (MSI) and abnormal cell cycle protein play an important role in HOTAIR oncogenic event. Diploid tumors with Microsatellite instability (MSI) have poor clinical outcome and respond worse [45, 46]. MMR defect seems to be related with sporadic-microsatellite instability (MSI) and Cell cycle proteins abnormal expression [47]. Our results indicate HOTAIR causes microsatellite instability (MSI) and abnormral expression of cell cycle related gene, e.g. CDK2, CyclinE, CDK4, CyclinD1, PCNA, ppRB, E2F1 in liver cancer stem cells. In fact, HOTAIR increased the expression of CyclinE, CyclinD1, CDK2, CDK4, ppRB, E2F1, PCNA which cause cell cycle loss-control and loss-regulation. For example, PCNA was shown to facilitate the switch between the replicative DNA polymerase with the low-fidelity polymerase eta to bypass UV-induced DNA lesions during replication [48].

## CONCLUSIONS

The present study depicts a novel provides evidence for HOTAIR to play tumorigenesis roles by downregulating SETD2 in liver cancer stem cells, which may have potential therapeutic significance. Alteration of the expression of lncRNAs HOTAIR may also mediate changes at an epigenetic level to affect gene expression and contribute to tumor aetiology. HOTAIR knockdown in combination with blocking DNA damaging reagents might represent a promising treatment strategy targeting tumors with over-activated HOTAIR. This understanding the novel functions of HOTAIR will help in the development of new liver cancer therapeutic approaches.

## MATERIALS AND METHODS

### Patients and tissue samples

Sixty-five cases of paired liver cancer tissues and their adjacent noncancerous liver tissues used for analysis were obtained from liver cancer patients who had undergone surgery. Informed consents were obtained from all patients. The liver cancer tissues and its adjacent noncancerous liver tissues were fixed in formalin before embedded in paraffin. All fresh liver cancer tissues and its adjacent noncancerous liver tissues snap frozen in liquid nitrogen within 30 minutes after surgery were available for Western blotting. All patients were diagnosed as liver cancer according to histological examination. These results were reviewed independently by at least three pathologists or clinicians.

### Human liver cancer stem cell line (hLCSC) sorting

CD133/CD44/CD24/EpCAM MicroBead Kits were purchased from Miltenyi technic (Boston, USA) and MACS^®^ Technology operation according to the manufacturer. In brief, centrifuge cell suspension at 300 × g for 10 minutes and resuspend human liver cancer cell line Huh7 cell pellet in 300 μl of buffer per 10^8^ total cells after aspirating supernatant completely. Add 100 μL of FcR Blocking Reagent per 10^8^ total cells and 100 μL of CD133/CD44/CD24/EpCAM MicroBeads per 10^8^ total cells. Mix well and incubate for 30 minutes in the refrigerator(2−8°C). Wash cells by adding 1−2 ml of buffer per 10^8^ cells and centrifuge at 300 × g for 10 minutes. Resuspend up to 10^8^ cells in 500 μL of buffer. Choose an appropriate MACS Column and MACS Separator according to the number of total cells and the number of CD133+/CD44+/CD24+/EpCAM+ cells.

### Cell lines and plasmids

Human liver cancer stem cell line (hLCSC) was maintained in Dulbecco's modified Eagle medium (Gibco BRL Life Technologies) or Minimum Essential Medium (MEM) (Gibco BRL Life Technologies) supplemented with 10% heat-inactivated fetal bovine serum (Gibco) in a humidified atmosphere of 5% CO_2_ incubator at 37°C. pGFP-V-RS, pCMV6-A-GFP, were purchased from Origene (USA);pGFP-V-RS-HOTAOR, pCMV6-A-GFP-HOTAIR, pcDNA3.1-STED2 were constructed by ourselves.

### Immunohistochemistry

Tissues were fixed with 4% paraformaldehyde, dehydrated, embedded in paraffin and sectioned at 4 μm. Sections were immunohistochemically stained using mouse anti-human monoclonal anti-SETD2, anti-PCNA(Santa Cruz, Biotech). As the secondary antibody, anti-mouse IgG (Horseradish peroxidase linked whole antibody from sheep, GE Healthcare Limited) was used at 100 × dilution. Staining was performed using 3, 3-diaminobenzidine (DAB) substrate kit for peroxidase according to the manufacturer's instructions (Vector Laboratories Inc) and counterstained with hematoxylin

### RT-PCR

Total RNA was purified using Trizol (Invitrogen) according to manufacturer's instructions followed by treatment with RNase-free DNase (QIAGEN). cDNA was prepared by using oligonucleotide (dT_18_), random primers, and a SuperScript First-Strand Synthesis System (Invitrogen). General PCR was performed in accordance with the manufacturers' protocols. Human β-actin was used as an internal control

### Western blotting

After being boiled for 10 minutes in the presence of 2-mercaptoethanol, samples containing cells or tissue lysate proteins were separated on a 10% sodium dodecyl sulfate-polyacrylamide gel electrophoresis (SDS-PAGE)and transferred onto a nitrocellulose membranes (Invitrogen, Carlsbad, CA, USA). NC membranes were blocked in 10% dry milk-TBST (20 mM Tris-HCl [PH 7.6], 127 mM NaCl, 0.1% Tween 20) for 1 h at 37°C. Following three washes in Tris-HCl pH 7.5 with 0.1% Tween 20, the blots were incubated with antibody (appropriate dilution) overnight at 4°C. Following three washes, membranes were then incubated with secondary antibody for 60 min at 37°C or 4°C overnight in TBST. Signals were visualized by enhanced chemiluminescence plus kit (GE Healthcare). Anti-mouse IgG (Horseradish peroxidase linked whole antibody was purchased from GE Healthcare Limited.

### Co-immunoprecipitation (IP)

Cells were lysed in 1 ml of the whole-cell extract buffer A (50 mM pH7.6 Tris-HCl, 150 mM NaCl, 1%NP40, 0.1 mM EDTA, 1.0 mM DTT, 0.2 mM PMSF, 0.1 mM Pepstatine, 0.1 mM Leupeptine, 0.1 mM Aproine). Five-hundred-microliter cell lysates was used in immunoprecipitation with antibody. In brief, protein was pre-cleared with 30 μl protein G/A-plus agarose beads (Santa Cruz, Biotechnology, Inc.CA) for 1 hour at 4°C and the supernatant was obtained after centrifugation (5,000 rpm) at 4°C. Precleared homogenates (supernatant) were incubated with 2 μg of antibody and/or normal mouse/rabbit IgG by rotation for 4 hours at 4°C, and then the immunoprecipitates were incubated with 30 μl protein G/A-plus agarose beads by rotation overnight at 4°C, and then centrifuged at 5000 rpm for 5 min at 4°C. The precipitates were washed five times × 10 min with beads wash solution(50 mM pH7.6 TrisCl, 150 mMNaCl, 0.1%NP-40, 1 mM EDTA) and then resuspended in 60 μl 2 × SDS-PAGE sample loading buffer to incubate for 5–10 min at 100°C. Then Western blot was performed with a another related antibody indicated in Western blotting.

### RNA immunoprecipitation (RIP)

Cells were lysed in 100 mM KCl, 5 mM MgCl_2_, 10 mM HEPES [pH 7.0], 0.5% NP40, 1 mM DTT, 100 units/ml RNase OUT (Invitrogen), 400 μM vanadyl-ribonucleoside complex and protease inhibitors (Roche), clarified and stored on at −80°C. Ribonucleoprotein particle-enriched lysates were incubated with protein G/A-plus agarose beads (Santa Cruz, Biotechnology,Inc.CA) together with primary antibody or normal rabbit IgG for 4 hours at 4°C. Beads were subsequently washed four times with 50 mM TRIS/HCl, pH 7.0, 150 mM NaCl, 1 mM MgCl_2_, and 0.05% NP-40, and twice after addition of 1 M Urea. IPs were digested with proteinase K (55°C; 30′) and mRNAs were isolated and then RT-PCR or qRT-PCR was performed according to the manufacturer's instructions.

### DNA pull down

Cells were lysed by sonication in HKMG buffer (10 mM HEPES, PH7.9, 100 mM KCl, 5 mM MgCl_2_, 100% glycerol, 1 mM DTT, and 0.5% NP40) containing protease and phosphatase inhibitors for the preparation of nuclear exact. Equal amount of cell nuclear extracts were precleared with Streptavidin-agarose Resin (Thermo) for 1 hours, and then were incubated with 1 μg biotinylated double-stranded-oligonucleotides and together with 10 μg poly(dI-dC) at 4°C for 24 hours. DNA-bound proteins were collected with the incubation with streptavidin-agarose Resin for 1 hour with gently shaking to prevent precipitation in solution. Following 5 washings of the resin bound complex with 0.5–1.0 ml of binding buffer, the samples were boiled and subjected to SDS-PAGE and Western blot analysis.

### Dual luciferase reporter assay

Cells (1 × 10^5^/well of a six-well plate) were transiently transfected by use of the Lipofectiamine™ 2000 (Invitrogen). After incubation for 48 h, the cells were harvested with Passive Lysis Buffer (Promega), and luciferase activities of cell extracts were measured with the use of the Dual luciferase assay system (Promega) according to manufacturer's instructions. luciferase activity was measured and normalized for transfection efficiency with Renilla luciferase activity

### Chromatin immunoprecipitation (CHIP) assay

Cells were cross-linked with 1% (v/v) formaldehyde (Sigma) for 10 min at room temperature and stopped with 125 mM glycine for 5 min. Crossed-linked cells were washed with phosphate-buffered saline, resuspended in lysis buffer, and sonicated for 8–10 min in a SONICS VibraCell to generate DNA fragments with an average size of 500 bp. Chromatin extracts were diluted 5-fold with dilution buffer, pre-cleared with Protein-A/G-Sepharose beads, and immunoprecipitated with specific antibody on Protein-A/G-Sepharose beads. After washing, elution and de-cross-linking, the ChIP DNA was detected by either traditional PCR (25–35 cycles).

### Nuclear run on assay

Nuclear run-on was performed by supplying biotin-probe to nuclei, and labeled transcripts were bound to streptavidin-coated streptavidin-agarose Resin [49]. The cells are chilled, and the membranes are permeabilized or lysed. The nuclei are then incubated for a short time at 37°C in the presence of nucleoside triphosphates (NTPs) and biotin labeled probe. The number of nascent transcripts on the gene at the time of chilling is thought to be proportional to the frequency of transcription initiation. To determine the relative number of nascent transcripts in each sample, the biotin labeled RNA is purified and hybridized to a membrane containing immobilized DNA from the gene of interest. The amount of biotin activity that hybridizes to the membrane is approximately proportional to the number of nascent transcripts.

### Restriction endonuclease analysis for DNA injury repair

We performed the modified method according to Li et al. [16]. In brief, the plasmid derived from pCMV6-AC frame was reconstructed containing BamHI site (GGATCC/CCTAGG) and mismatch EcoRI site (GAATTC/GAAAAG). The mammalian cells can be harvested less than 48 hours after transfection and any contamination with plasmid which has not yet been into the cells is not produced. By doing so, the mammalian cell pellets are completely rinsed. Because plasmid can not replicate or amplify in mammalian cells, the plasmid shold be recovered at the first time. DNA is extracted from cytosolic lysates using plasmid extracted kit. After the plasmid DNA is prepared, the plasmid is transformed into the bacterial DH5α to screen and amplify the plasmid. Restriction endonuclease analysis with BamHI and EcoRI is performed using the extracted plasmid.

### MSI detection through dot blot (Slot blot)

Dot blots can only confirm the presence or absence of a biomolecule or biomolecules which can be detected by the DNA probes. Various Biotin labling MSI probes (Biotin-MSIs) added individually to the wells where a vacuum sucks the water (with NaOH and NH_4_OAc) from underneath the membrane (nitrocellulose) as a dot and then is spotted through circular templates directly. The cells DNA is quantified and equal amounts are aliquoted into tubes. These are denatured (NaOH and 95°C) and can be hybridized with the membrane to allow for the detection of variation between samples. The signal can be detected by anti-Biotin Western blotting.

### Cell transfection and stable cell lines

hLCSC cells were transfected with pCMV-A-GFP, pCMV6-A-GFP-HOTAIR, pGFP-V-RS, pGFP-V-RS-HOTAIR using transfection reagent lipofectamine^R^ 2000 (Invitrogen) according to manufacturer's instructions respectively. For screening HOTAIR overexpression or knocked down stable cell lines, using 1–2 μg/ml Puromycin (Calbiochem) or 2 mg/ml G418 (Invitrogen) was added the following day.

### Cells proliferation CCK8 assay

To describe growth curves, cells were synchronized in G0 phase by serum deprivation and then released from growth arrest by reexposure to serum, and then cells were grown in complete medium for assay. In brief, cells at a concentration 4 × 10^3^ were seeded into 96-well culture plates in 100 μl culture medium containing 10% heat-inactivated fetal calf serum(FCS). Before detected, add 10 μg/well cell proliferation reagent CCK8 (Calbiochem) and incubate for 2–4 hours at 37°C and 5% CO_2_ according to the manufacturer instruction. Cells growth curve was based on the corresponding the normalized values of OD450 and each point represents the mean of three independent samples.

### Cell colony-formation efficiency assay

1 × 10^3^ cells were plated in 10 cm dish and incubated in a humidified atmosphere of 5% CO_2_ incubator at 37°C for 10 days. For visualization, colonies were stained with 0.5% Crystal Violet (sigma) in 50% methanol and 10% glacial acetic acid. Colonies were counted using a dissecting microscope by MacBiophotonics Image J.

### BrdU staining

80% confluent cells were cultured for 24 hour before treatment with 10μl BrdU (Roche) for 4 hours. Immunofluorescent staining with an anti-BrdU antibody was performed according to the manufacturer's instructions (Becton Dickinson). BrdU positive cells from ten random chosen fields of at least three independent samples were counted.

### Xenograft transplantation *in vivo*

Four-weeks male athymic Balb/C mice were purchased from Shi laike company (Shanghi, China) and maintained in the Tongji animal facilities approved by the China Association for accreditation of laboratory animal care. The athymic Balb/C mouse was injected at the armpit area subcutaneously with suspension of 5 × 10^7^ transfected cells in 100 μl of phosphate buffered saline. The mice were observed over 4 weeks, and then sacrificed to recover the tumors. The wet weight of each tumor was determined for each mouse. A portion of each tumor was fixed in 4% paraformaldehyde and embedded in paraffin for histological hematoxylin-eosin (HE) staining and anti-PCNA immunochemical staining. The use of mice for this work was reviewed and approved by the institutional animal care and use committee in accordance with China national institutes of health guidelines.

### Statistical analysis

The significant differences between mean values obtained from at least three independent experiments. Each value was presented as mean ± standard error of the mean (SEM) unless otherwise noted, with a minimum of three replicates. The results were evaluated by SPSS20.0 statistical soft (SPSS Inc Chicago, IL) and Student's *t*-test was used for comparisons, with *P* < 0.05 considered significant.
